# Experimental and Numerical Studies of Low-Profile, Triangular Grid-Stiffened Plates Subjected to Shear Load in the Post-Critical States of Deformation

**DOI:** 10.3390/ma12223699

**Published:** 2019-11-09

**Authors:** Łukasz Święch

**Affiliations:** Faculty of Mechanical Engineering and Aeronautics, Rzeszów University of Technology, al. Powst. Warszawy 8, 35-959 Rzeszow, Poland; lukasz.swiech@prz.edu.pl

**Keywords:** thin-walled structures, integral structures, stiffened plate deformations, triangular grid stiffened structure, post-critical deformations, isogrid structures, aerospace structures, aircraft load-bearing structures, nonlinear numerical analyses, digital image correlation

## Abstract

Constant developments in manufacturing technology have made it possible to introduce integrally stiffened elements into load-bearing, thin-walled structures. The application of thin-walled elements with integral stiffeners potentially increases buckling and critical loads to maintain the mass of the structure and lower production costs. This paper presents the results of experimental investigations and numerical Finite Element Modelling (FEM) analyses of low-profile, isosceles grid stiffened, aluminium alloy plates subjected to pure shear load. Conducted research included analysing buckling and post-buckling states of deformation, taking into account both geometrical and physical nonlinear effects. Use of the Digital Image Correlation (DIC) system during the experimental tests created representative equilibrium pathways and recorded displacement field distributions over the plate surface. The model was initially validated against the experimental results. The results for the stiffened plate were compared to the reference structure in the form of a smooth plate with equivalent mass. Comparative analyses included examining the displacement fields and stress efforts over the plates. The stiffening configuration under examination increased the critical buckling load by 300% in comparison to the unstiffened structure with the same mass. Obtained results also indicate potential problems with areas of concentrated stress in the case of an incorrect geometry design near to the boundary conditions.

## 1. Introduction

Thin-walled, stiffened panels are the most common load-bearing elements in the design of light-weight structures in many areas of engineering. In classic solutions, stiffening elements manufactured as separate details are combined with the relatively thin skin. Such constructions arranged in the spatial structures, called semi-monocoque, are able to withstand design loads with a high load-bearing capacity [1,2,3].

The main technique to join stiffening elements, such as stringers and ribs, to the skin is riveting, but the constant pursuit to lower the mass of the structure forces the search for new solutions. One possible alternative is to replace the traditional joining process by new technologies such as friction stir welding [4,5,6,7]. On the other hand, the constant development of new production methods such as numerically controlled machining tools allows also for the creation of so-called integral constructions, in which both the skin and stiffeners are made of one piece of material. Such an approach makes it possible to reduce the weight of the structure, by eliminating elements connecting individual parts, and also has an influence on increasing the damage tolerance [8,9]. Application of integral load-bearing structures also makes it possible to introduce skins with so-called sub-stiffening. The idea is based on the introduction of low-profile, integral stiffening elements to improve the buckling resistance without changing the mass of the structure. The positive impacts of such a solution, in the case of prismatic sub-stiffening on buckling and limit loads of thin-walled panels, have been under consideration by means of experimental and numerical studies [10,11,12].

Among many types of possible integral stiffening methods, the use of the grid structures comprising a lattice of rigid, interconnected ribs seems the most promising with use of modern Computerized Numerical Control (CNC) machines and the potential application of 3D printing in production. Grid structures with ribs running in three directions, forming equilateral triangles, are called isogrids. These structures are characterized by a very high ratio of stiffness and strength to their weight [11,13,14]. Recurrence in stiffening geometry also has a great influence on the damage tolerance of the structure. Metallic grid structures have mostly been used in the design of space rockets and still are under consideration in many industrial initiatives (e.g., SpaceX and Boeing spaceships or Airbus A330neo wing parts). It could be also assumed that the development of spatial printing technology will increase the interest of such a structural solution to produce geometrically complicated structures [15].

The first extensive study on this type of structure was commissioned by NASA in 1973 [13], where the smeared stiffened method was proposed for simplified analytical calculations. The theory of this method is based on the assumption that the stiffened structure can be treated as an equivalent orthotropic one by smearing the stiffness of the stiffeners into the plate [16,17].

Over the years, a number of publications have been denoted to smeared stiffened methods for both metal and composite grid structures. This method is under constant development, and it is used for predictions of global and local buckling loads and failure modes. Wang et al. [18] presented a numerical-based method with the use of asymptotic homogenization for obtaining equivalent stiffness coefficients for grid-stiffened composite shells. Jaunky et al. [19] formulated an improved smeared stiffener theory with the introduction of skin-stiffener interaction effects for panels under compression. A similar interaction was proposed by Xu et al. [20] with applications to different types of grids by introducing a vector parameter related to the stiffener configuration. Wang and Abdalla [21] presented a method to obtain panel properties corresponding to classical lamination theory by matching the strain energy of stiffened and equivalent unstiffened panel cells for global buckling analyses. In the above-mentioned papers, calculated buckling loads for several stiffener configurations were compared with FEM results. Luan et al. [22] proposed improvements to the simplified smearing technique for modelling vibrations of stiffened plates. Due to its simplifications, the smeared stiffened method has the most significant role in the preliminary design. To obtain more accurate results, including the local effects in the nodes of the grid and taking into account nonlinear effects, the application of more sophisticated numerical methods is required. Huybrechts and Tsai [23] developed a computer code to predict grid structure deformation behaviour and failure by determining, among others, grid structure strengths and the effects of missing ribs. Kopecki et al. [24,25] investigated the effects of post-critical deformations and cut-outs on the strength of thin-walled, semi-monocoque structures subjected to shear. In the case of geometrically complex structures, it may also be advisable to adopt methods developed for composite structures, such as optimization with use of reanalysis [26,27] or analysis of variable stiffness plates based on a reduced order model [28].

This paper presents results of experimental and numerical investigations of post-buckling states of deformation of a low-profile, isosceles grid stiffened plate. The plate was treated as the skin of semi-monocoque structures and was subjected to pure shear loading conditions. In the proposed approach, the panel under consideration was a sub-stiffened skin element, and it was allowable for it to lose stability in the operational load range of the structure. Experimental studies and numerical FEM analyses were carried out. The main scope of the research was to identify the potential benefits of the integral stiffened element under consideration in comparison to a smooth plate with equivalent mass. The secondary objective was to analyse the possibility of exact FEM modelling of such structures with comparisons to the experimental results obtained with the use of the Digital Image Correlation (DIC) system.

## 2. Theoretical Basis

**Figure 1:**
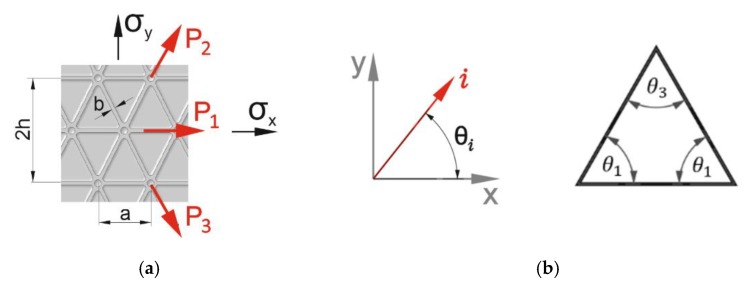
Forces in grid (**a**) and angle definitions (**b**).

The basic analytical method to calculate triangularly grid-stiffened structures is the smeared stiffened method. The structure under consideration (Figure 1a) consisted of ribs and skin, and it may be treated as layered material with appropriate elastic constants for each element. It can be assumed that ribs are in a state of uniaxial stress, and the relation of Hooke’s law can be adopted for elements in the gridwork. Internal strains are determined by the stress resultants and couples in the composite construction. From strains, the stresses in the elements may be determined. Stresses in bars are dependent upon its orientation, and for the skin (Figure 1b), they depend upon the orientation of the normal of the plane upon which the stresses are assumed to act.

With the use of the strain transformation Equation (1), the relation between uniaxial bar strain $\varepsilon_{i}$ and *x*, *y* coordinate grid strains can be obtained:

(1) $$\varepsilon_{i}=\varepsilon_{x}\cos^{2}\theta_{i}+\gamma_{xy}\sin\theta_{i}\cos\theta_{i}+\varepsilon_{y}\sin^{2}\theta_{i},\;i=1,2,3$$

taking into account that uniaxial bar loads $P_{i}$ are as given Equation (2):

(2) $$P_{i}=bE\varepsilon_{i}$$

Grid stress components in the *x* and *y* directions can be found by dividing the grid load by periodic lengths of the grid Equation (3):

(3) $$\sigma_{x}=\frac{2P_{1}+\left(P_{2}+P_{3}\right)\cos\theta_{1}}{2h},\;\sigma_{y}=\frac{\left(P_{2}+P_{3}\right)\sin\theta_{1}}{a},\;\tau_{xy}=\frac{\left(P_{2}-P_{3}\right)\sin\theta_{1}}{2h}$$

By comparing Equations (1) and (3) with the Hook’s law relations for an isosceles triangle grid, the law for orthotropic structure in-plane stress can be expressed as Equation (4):

(4) $$\left(\begin{array}{c} \sigma_{x}\\ \sigma_{y}\\ \tau_{xy} \end{array}\right)=\left(\frac{b}{2h}E\right)\left[\begin{array}{ccc} 2\left(1+\cos^{3}\theta_{1}\right) & 2\sin^{2}\theta_{1}\cos\theta_{1} & 0\\ \frac{4h}{a}\cos^{3}\theta_{1}\sin^{2}\theta_{1} & \frac{4h}{a}\sin^{3}\theta_{1} & 0\\ 0 & 0 & 2\sin^{2}\theta_{1}\cos\theta_{1} \end{array}\right]\left(\begin{array}{c} \varepsilon_{x}\\ \varepsilon_{y}\\ \gamma_{xy} \end{array}\right)$$

Taking into account compliance *S* and stiffness *Q* matrices for in-plane stress, the engineering constants are denoted by Equation (5):

(5) $$S=\left[\begin{array}{ccc} \frac{1}{E_{1}} & -\frac{\nu_{12}}{E_{1}} & 0\\ -\frac{\nu_{21}}{E_{2}} & \frac{1}{E_{2}} & 0\\ 0 & 0 & \frac{1}{G_{12}} \end{array}\right],\;Q=\left[\begin{array}{ccc} \frac{E_{1}}{1-\nu_{12}\nu_{21}} & \frac{\nu_{12}E_{1}}{1-\nu_{12}\nu_{21}} & 0\\ \frac{\nu_{21}E_{2}}{1-\nu_{12}\nu_{21}} & \frac{E_{2}}{1-\nu_{12}\nu_{21}} & 0\\ 0 & 0 & G_{12} \end{array}\right]$$

A comparison of Equations (4) and (5) enables to express engineering constants for isosceles triangle grid-stiffened structures as Equation (6):

(6) $$E_{1}=\left(\frac{b}{2h}E\right)2=\frac{b}{h}E,\;E_{2}=\left(\frac{b}{2h}E\right)\frac{4h}{a}\frac{\sin^{3}\theta_{1}}{1+\cos^{3}\theta_{1}}=\left(\frac{b}{a}E\right)\frac{\sin^{3}\theta_{1}}{1+\cos^{3}\theta_{1}},\;\nu_{12}=\left(\frac{2h}{a}\right)\frac{\cos^{2}\theta_{1}\sin\theta_{1}}{1+\cos^{3}\theta_{1}},\;\nu_{21}=\left(\frac{a}{2h}\right)\frac{1}{\tan\theta_{1}},\;G_{12}=\left(\frac{b}{h}E\right)\cdot 2\sin^{2}\theta_{1}\cos\theta_{1}$$

where $E_{1}$, $E_{2}$ are the longitudinal and transverse Young’s modulus, $G_{12}$ is the in-plane shear modulus, and $\nu_{12}$, $\nu_{21}$ are the Poisson’s ratio.

If a structure can be idealized by a plate or shell, the loading on the surface is considered to be resistant to stress and stress couples obtained by integrating the stresses and moments in the thickness direction.

## 3. The Object of the Study

**Figure 2:**
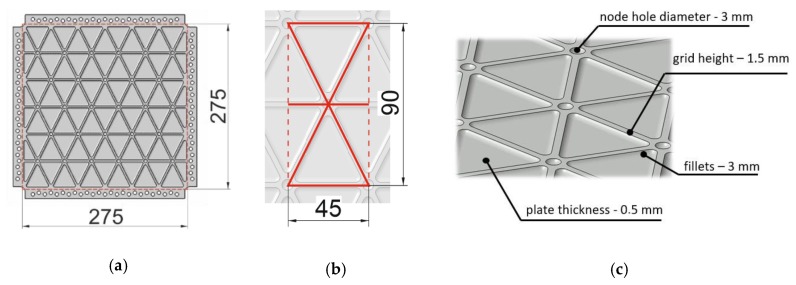
The geometry of the tested plate: (**a**) overall dimensions, (**b**) grid geometry, (**c**) detailed dimensions of the grid components.

Experimental studies were carried out on 306 × 306 mm square plates with a test field of 275 × 275 mm (Figure 2a), machined from 2 mm thick duralumin 2024 T3 plates with the use of a CNC milling machine (Haas Automation Inc., Oxnard, CA, USA) and a specially prepared vacuum table. The use of a table was necessary to ensure a high geometrical tolerance of the manufacturing process. Such a test structure was prepared as an integrally stiffened plate with a periodic isosceles triangle grid of ribs of dimensions 45 mm by 90 mm (Figure 2b). Ribs were 2 mm thick and 1.5 mm high. In every node in the grid, a hole with a diameter of 3 mm was drilled. The skin of structure, the areas between ribs, had a thickness of 0.5 mm (Figure 2c).

**Figure 3:**
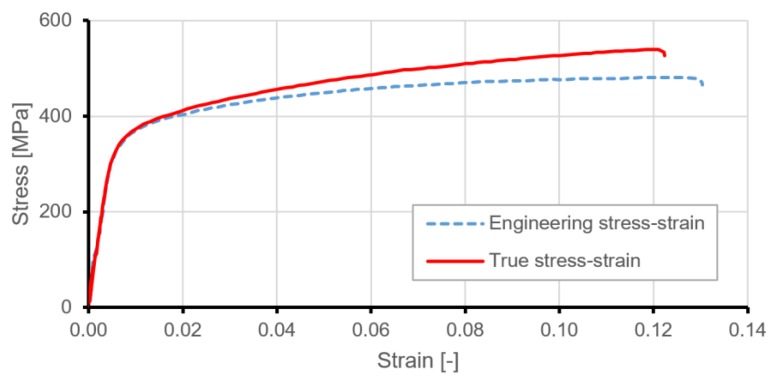
Nominal stress–strain curves for 2024 aluminium alloy.

As a reference structure with equivalent mass, a smooth plate with a thickness of 0.75 mm and identical overall dimensions was chosen. All subsequent considerations and their results were conducted in relation to the reference structure. Figure 3 presents nominal relations between stress and strain for duralumin 2024 T3 used in the manufacturing of tested samples.

## 4. Experimental Test Stand

**Figure 4:**
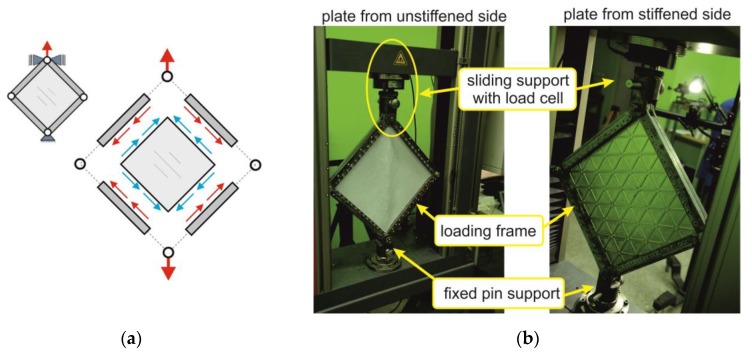
Experimental setup: (**a**) loading conditions, (**b**) plate mounted in the test area of the strength machine.

During the experimental research, the plate was subjected to pure shear loading conditions. The plate was mounted in a steel, hinged frame, which transferred the vertical force from the strength machine into the pure shear on the edges of the plate (Figure 4). Experimental tests were conducted with the use of a universal strength testing machine Zwick Z050 (ZwickRoell GmbH &Co.KG, Ulm, Germany) with force level control. During research, the plate was subjected to loads up to 40 kN, which resulted in both post-critical and plastic deformations.

A three-dimensional Digital Image Correlation (DIC) system, ARAMIS (GOM GmbH, Braunschweig, Germany), was used during the experiment. ARAMIS is a non-contact and material-independent measuring system that captures and analyses deformations of the measured object in consequent load steps over time [29,30]. Measurements with the use of the ARAMIS system consisted of taking a series of images of the specially prepared object. Preparation involved covering the measurement surface with a stochastic pattern of black spots. Two stereo-metrically arranged digital cameras were used to record the displacements of spots related to strain changes due to the increase in loading. The quality of the pattern also had a significant influence on the accuracy of results.

During the test, displacement fields were recorded on the unstiffened side of the plate at a frequency of 1 Hz. The analogue electrical connection between testing devices allowed to assign the acting load information in every step of deformation.

Deformations of the stiffened plate captured by the DIC system is available as the supplementary file [Video S1].

**Figure 5:**
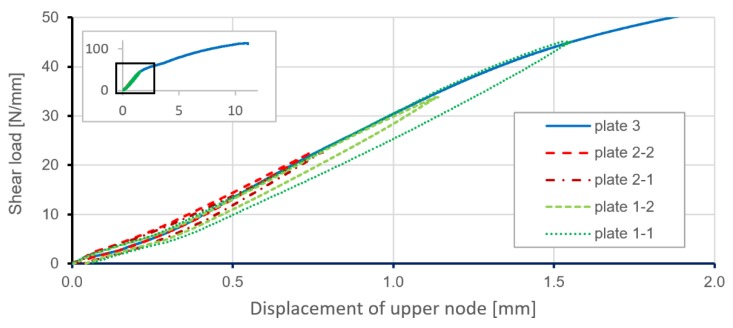
Shear load versus displacement of the upper node of the loading frame obtained for stiffened plates. Results from the strength test machine.

Experimental tests were performed on three geometrically identical specimens. Figure 5 presents relations between shear load and displacement of the upper node of the loading frame for a series of different load levels applied to the plates. Only one plate (plate 3) was loaded to failure in the static test, and this result was used to later validate the numerical model. Obtained results for all specimens proved the repeatability of plate behaviours in the range of elastic strain.

Results of the experimental investigations are presented in Section 6 together with numerical analysis results.

## 5. Numerical Modelling

A finite element model of the considered structure was developed with the use of ABAQUS/Standard commercial software (6.13, DassaultSystèmes, Vélizy-Villacoublay, France). The problem under consideration was nonlinear, both physically and geometrically, and it was purposeful to use as simple an FEM model as possible. In order to save computing time, and to maintain the ability to simulate the local behaviour of the structure, the shell element was used.

### 5.1. Material Modeling

Because deformations in the range of operational loads are related to elastic strain, simplified material models were used in the paper. The material of the frame was assumed as perfect linear elastic steel, and for the plate, an aluminum alloy model for elastoplastic material was used (Table 1).

### 5.2. Boundary Conditions

The steel frame was modelled by beam B31 elements. Frame members were connected at the corners by means of a HINGE connection type. Bolt connections between the plate and frame were modelled with the use of MPC elements with the TIE constraint. To reproduce experimental boundary conditions, sliding and fixed supports were applied at the upper and bottom nodes of the frame, respectively.

### 5.3. Mesh and Elements

**Figure 6:**
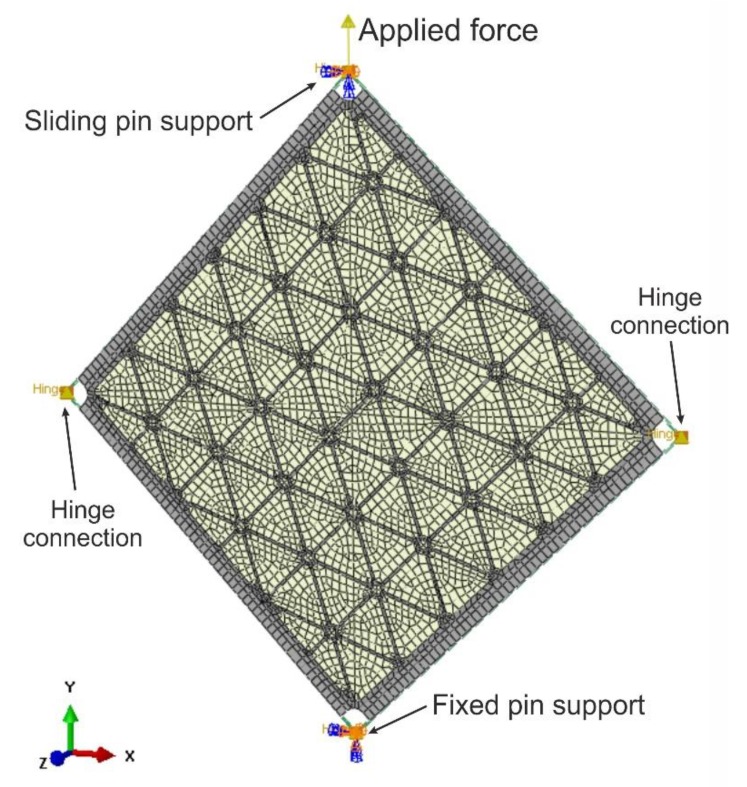
FEM model and boundary conditions.

The plate was modelled with the use of the four (S4R) and three (S3R) nodes, reduced integration and large-strain formulations standard library shell elements [31]. To reproduce the geometry of the plate and triangularly arranged ribs, all elements were placed in one plane, and various thicknesses of plate and rib areas were modelled by appropriate geometrical properties (Figure 6). Such modelling allowed to reconstruct fillets between ribs in the nodes of the grid, but information about fillets in the direction normal to the plate (i.e., between plate and ribs) was missed. For a comparison of results, a second model consisting of a smooth plate with equivalent mass was developed.

**Figure 7:**
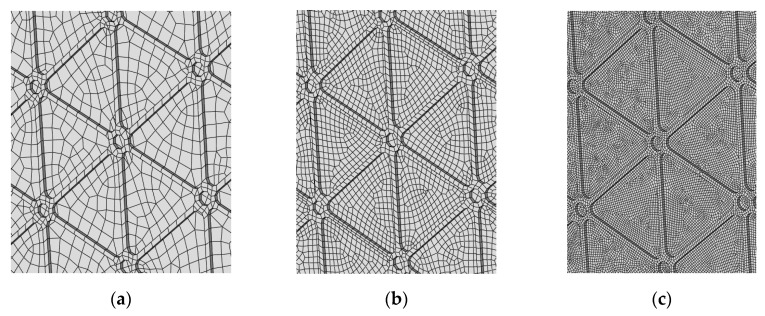
Mesh sizes chosen to perform the convergence study. Characteristic element length: (**a**) 5 mm, (**b**) 2.5 mm and (**c**) 1 mm.

**Figure 8:**
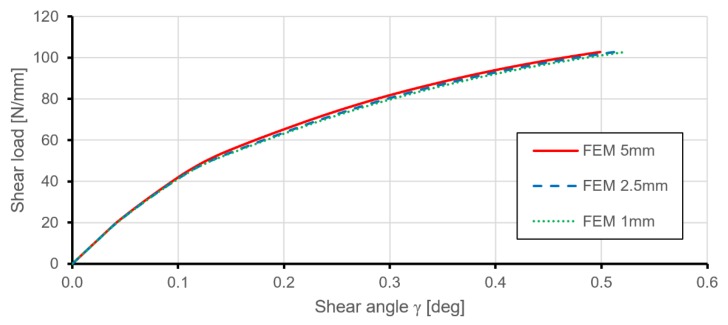
Comparison of results of shear load versus shear angle obtained from FEM for the stiffened plate in the convergence study.

A convergence study of critical buckling loads and post-buckling behaviours of the structure was performed to define the appropriate mesh size. Figure 7 presents the element mesh grids used in the convergence study, and Figure 8 presents the numerical results obtained for these three models. The selected mesh had an average element size of 2.5 mm and consisted of 17,947 elements.

### 5.4. Validation

Nonlinear numerical analyses were carried out with the use of modified prognostic Newton–Raphson procedures with standard load corrections [32,33,34,35]. The first step of analysis for both stiffened and smooth plates involved a simplified, linearized analysis of stability. Results of the linear buckling analyses were aimed at predicting the critical load and shape of the deformation field, which was used next as the initial imperfection of the structure. Nonlinear analyses were performed for the models with geometrical imperfections introduced as the first buckling mode shape, with small amplitudes of 1%, 5%, and 10% of the plate thickness and one model without initial imperfections. Results of FEM analyses in comparison to the experimental investigation results are presented in the subsequent section.

## 6. Results

The discussion of results has been divided into two main parts. The first one compares the experimental and numerical results for a low-profile, triangular grid stiffened plate and is aimed at validating the used FEM model. The second part compares results for numerical studies of the stiffened plate and smooth one with equivalent mass to determine the impact of introduced integral ribs on the stress level and deformations of structures, including post-critical behaviours.

### 6.1. Comparison of FE Results and the Experiment for the Stiffened Plate

**Figure 9:**
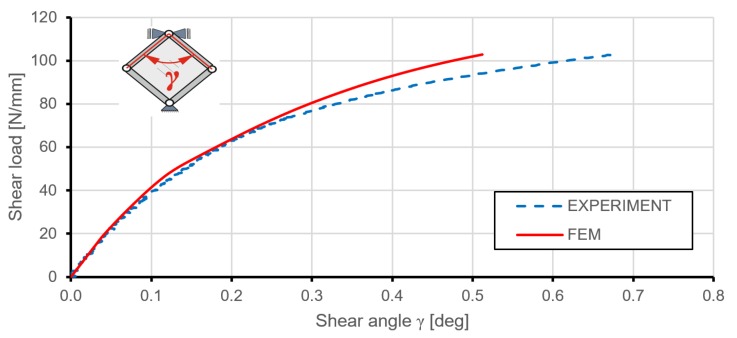
Shear load versus shear angle obtained for stiffened plates.

In order to illustrate the behaviour of the structure in the post-critical states of deformation, so-called representative equilibrium pathways were created. Figure 9 presents a comparison of relations between shear edge loading and the consequent shear angle for experimental and numerical results. The numerically predicted global, in-plane stiffness was in good agreement with the experiment to a load level of about 75 N/mm. Above that value of the acting load, the FEM model had a higher in-plane stiffness, although it must be noted that at a load level of 36 N/mm, the numerical results revealed initial plastic deformation of the structure. Differences in deformations probably were due to geometric simplifications and the used mechanical properties of the plate material. FEM results did not indicate significant differences for models with and without geometrical imperfections.

**Figure 10:**
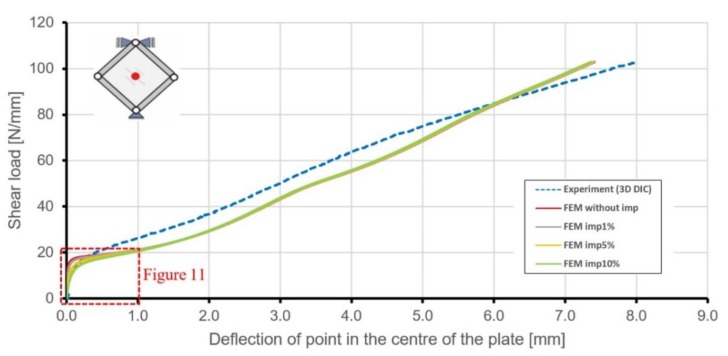
Comparison of shear load versus central point deflection curves, obtained for the stiffened plate by numerical calculations and experimental investigations (imp means geometrical imperfection).

Because of the plate lost stability during the experiment, a second equilibrium path was chosen (Figure 10). The relationship between the acting load and deflection of the point in the geometrical centre of the plate proved to be more reliable in the case of deflection of the structure. Differences between numerical and experimental results were more significant in this case. In every step of deformation below, about 85 N/mm, the FEM model had higher out-of-plane displacements. Such a situation can be explained by simplifications in the geometry of numerical models (e.g., lack of fillets between skin and ribs), which leads to lower out-of-plane stiffness of the structure and possible greater stress concentrations areas in the nodes of the triangular grid of ribs.

**Figure 11:**
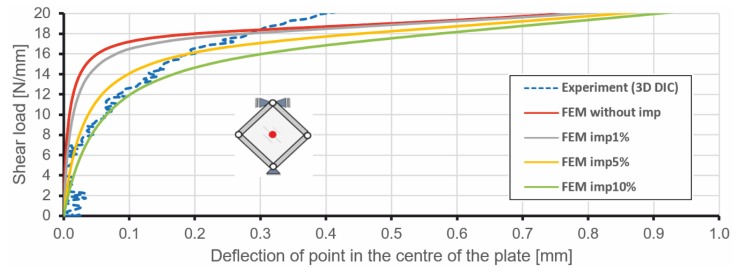
Load versus central point deflection obtained for the stiffened plate by numerical calculations and experimental investigations.

Initial geometrical imperfections introduced to the FEM model resulted in different behaviours of the stiffened plate for low levels of loading, but from the level of about 20 N/mm, all curves obtained from numerical analyses were almost identical (Figure 11). Conducted numerical analyses have shown, as might be expected, that the increase in initial geometrical imperfections leads to milder equilibrium paths in the range of loads connected with the loss of stability.

**Figure 12:**
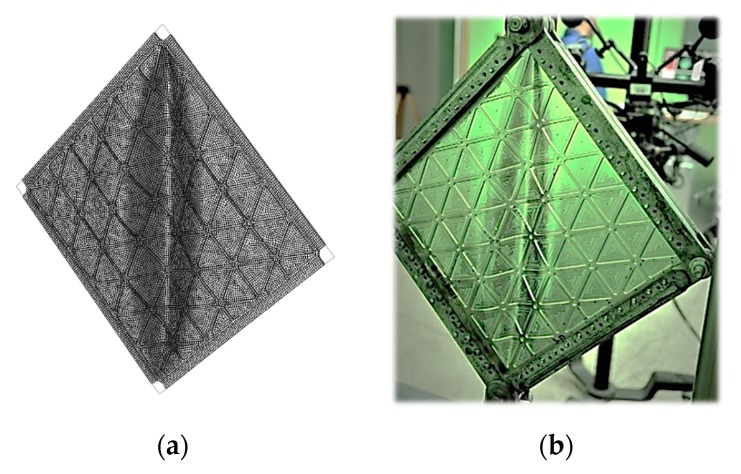
Qualitative comparison of the deflection field at the maximum load. (**a**) The numerical result, (**b**) the experimental result.

**Figure 13:**
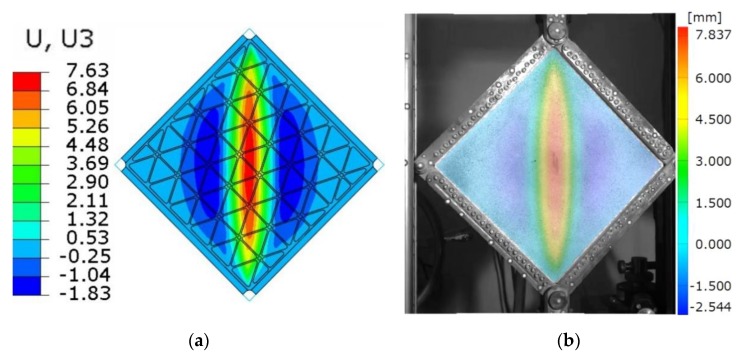
Quantitative comparison of the deflection field at the maximum load. (**a**) The numerical result, (**b**) the experimental DIC result.

Figure 12 presents a qualitative comparison of numerically and experimentally obtained deformation fields captured at the level of maximum load. Figure 13 presents the fields of deflection of the structure obtained as the results of numerical analysis and experimental investigation. It is worth to note that, besides the above-mentioned differences in the value of deflections, the overall deformation shape of the finite model converged with experimental results.

**Figure 14:**
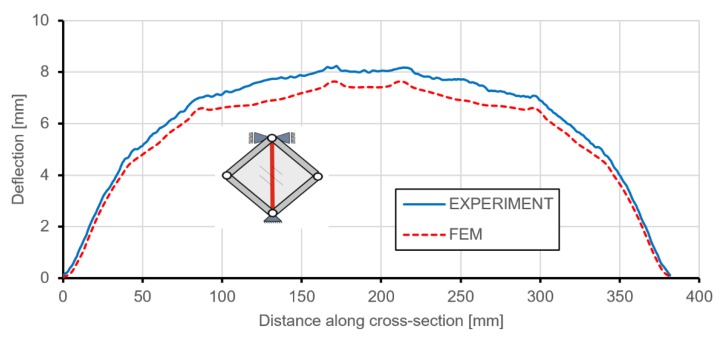
Deflection of a cross-section through the vertical diagonal at the level of maximum load.

**Figure 15:**
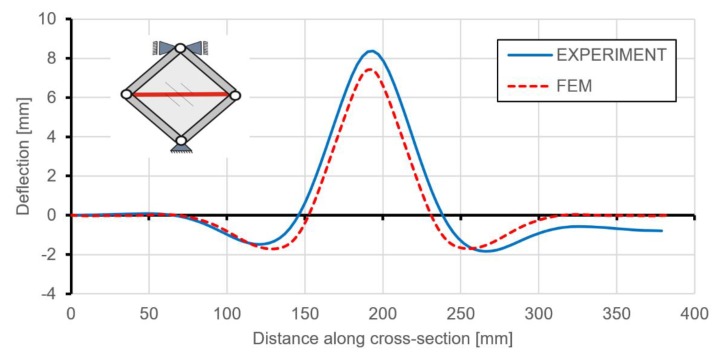
Deflection of a cross-section through the horizontal diagonal at the level of maximum load.

**Figure 16:**
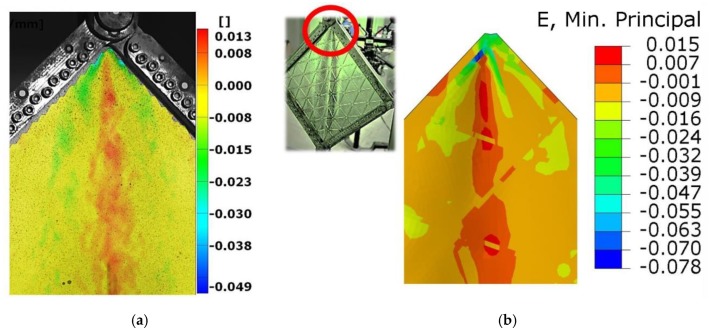
Comparison of the minimum principle strain distribution at the maximum load. (**a**) Experimental DIC result, (**b**) numerical result.

Use of the DIC scanner ARAMIS made it also possible to quantitatively compare any type of deformation. Figure 14 and Figure 15 present the deflections of a cross-section through the vertical and horizontal diagonal at the maximal load level achieved in the experimental study. The vertical cross-section, except for the overall deflection, also shows local deformations between ribs in the range of plastic deformations. This phenomenon is documented in Figure 14, where rib areas are represented by peaks and between them it is possible to observe different deformations in the form of buckling of the skin. Figure 16 presents strain fields in the area of the upper node of the test frame, recorded by the DIC system and obtained by FEM analyses, caused by the maximum level of the acting load. A comparison of the above similarities proved that the FEM model used provided results with a sufficient level of convergence.

### 6.2. Comparison of FE Results for Stiffened and Smooth Plates

Numerical analyses carried out made it possible to compare deformations and stress states of the stiffened plate and the smooth plate with a thickness 0.75 mm and equivalent mass.

**Figure 17:**
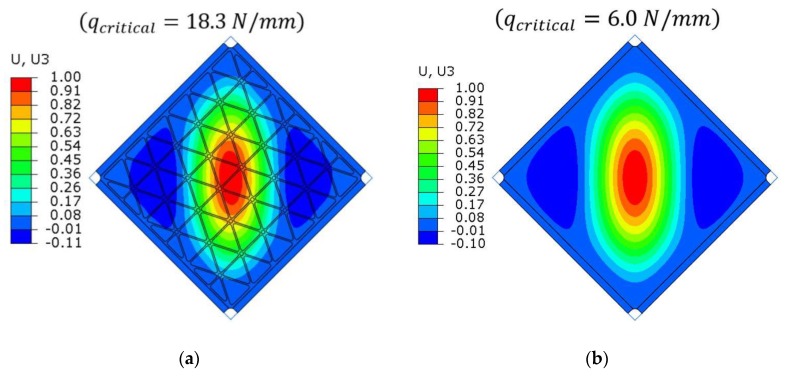
Results of linearized buckling FEM analyses. Normalized deflection fields for the first buckling modes for (**a**) the stiffened plate and (**b**) the smooth plate. The values given in parentheses mean the critical shear edge load corresponding to the first buckling mode. The shape of deformation used as geometrical imperfections in the first step of nonlinear FEM analyses.

The numerically predicted critical load for the stiffened plate was higher by 300% in comparison to the smooth plate. The obtained value was equivalent to the plate with a thickness of 2 mm, but the mass of such an element would be higher by 166%. The shapes of first buckling mode obtained by the linearized solution, in a qualitative manner, were the same for both plates (Figure 17).

Similar to the case of the stiffened plate, for the smooth plate the initial imperfection was introduced to the input file of the FEM model. The shape of imperfection was chosen as the first buckling mode (Figure 17) with a maximal value of initial deflection equal 0.05 mm, which was 6.67% of the smooth plate’s thickness. Because all finite elements and their centroids were in one plane for the smooth plate, the existence geometric disorder was necessary to allow the loss of stability in the case of nonlinear analyses. This was in opposition to the stiffened plate, where the presence of initial geometrical imperfections in numerical analyses had a significant influence on deformations, but it was not a necessary condition causing the loss of stability of the structure.

**Figure 18:**
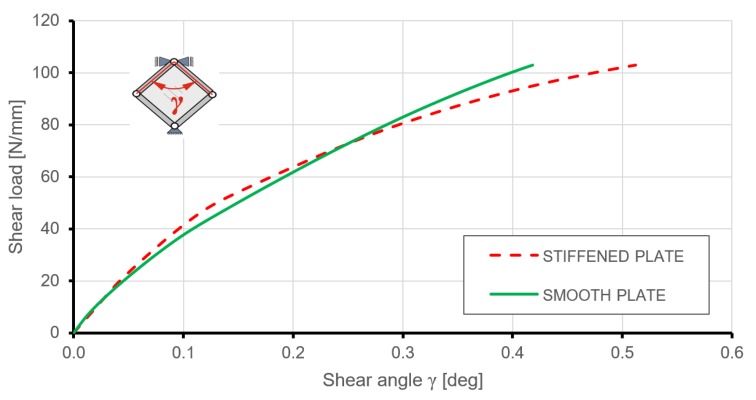
Comparison of shear load versus shear angle obtained from FEM for stiffened and smooth plates.

Figure 18 presents a comparison of the shear angle change due to load increases for stiffened and smooth plates. Up to a load level of about 70 N/mm, the equilibrium paths were very similar, both qualitatively and quantitatively; therefore, the in-plane stiffness plate with gridded ribs did not manifest a significant advantage over the smooth plate with equivalent mass.

**Figure 19:**
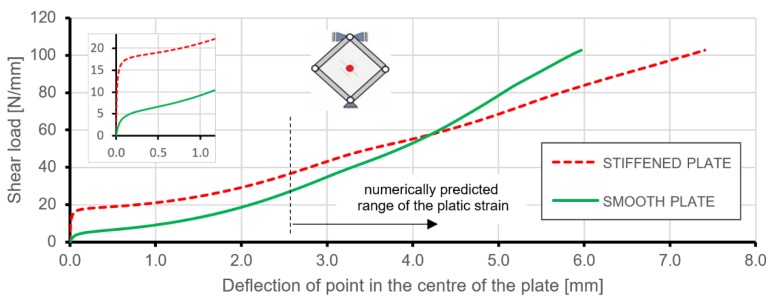
Comparison of shear load versus deflection obtained from FEM for stiffened and smooth plates.

Comparing equilibrium paths expressed by the relation between the deflection of points in the geometrical centre of the plates and acting loading gives a more complete understanding of the deformation behaviour of the analysed structures (Figure 19). Similarly to the predictions of linearized buckling, the geometrically linear range of deformation for the stiffened plate ended at a much higher load level than in the case of the smooth plate. The absence of steep changes in the equilibrium paths between pre- and post-buckling deformations were caused by the introduction of geometrical imperfections to the numerical plate models.

Analysis of the charts presented in Figure 19 makes it possible to compare the out-of-plane stiffness of plates under consideration. In the range of loading up to 55 N/mm, the stiffened plate deflection was much lower than in comparison to the smooth plate with equivalent mass. Nonlinear FEM analyses, just like linearized buckling, showed a significant impact of triangular stiffening on the critical load of the plate. An enlarged area in Figure 19 presents the transitions from pre- to post-buckling states of deformation. Smooth transitions, instead of sudden changes in equilibrium paths, were caused by the introduced initial geometrical imperfection. Despite this fact, critical loads were obtained at a level close to the simplified buckling analyses.

FEM analyses allowed also to determine the load limit, which caused plastic strain in the studied structures. First, permanent deformation occurred for almost identical deformation values in terms of the deflection for both structures, which were about 2.58 mm for the stiffened plate and 2.61 mm for the smooth plate. However, load levels related to the exhibition of plastic strain were about 37 N/mm and 28 N/mm for stiffened and smooth plates, respectively. These pointed out differences in the values of limit loads proves that the introduction of integral stiffening to the plate increased the load level by a little over 31% without an increase in the mass of the structure. The dotted line in Figure 19 represents the numerically obtained beginning of the range of the plastic strains in the structures. Analyses of deformations and stress states in this range may be affected by the errors resulting from the simplified material and geometrical models of the stiffened plate used in FEM calculations.

**Figure 20:**
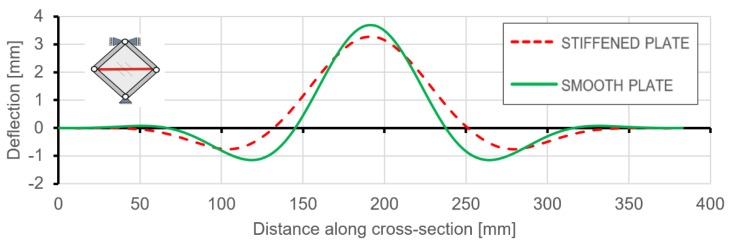
Comparison of horizontal cross-section deflections at the load level causing the start of plastic deformation (q = 37 N/mm).

**Figure 21:**
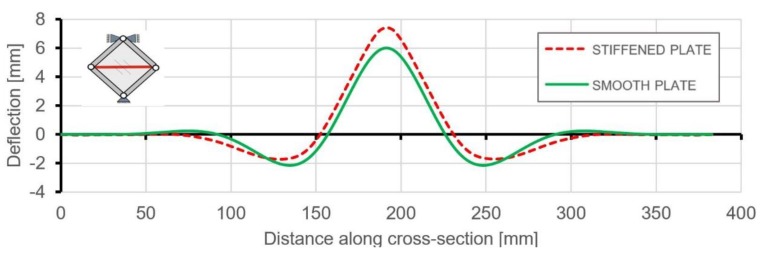
Comparison of horizontal cross-section deflections at the maximum load level equal to 103 N/mm.

**Figure 22:**
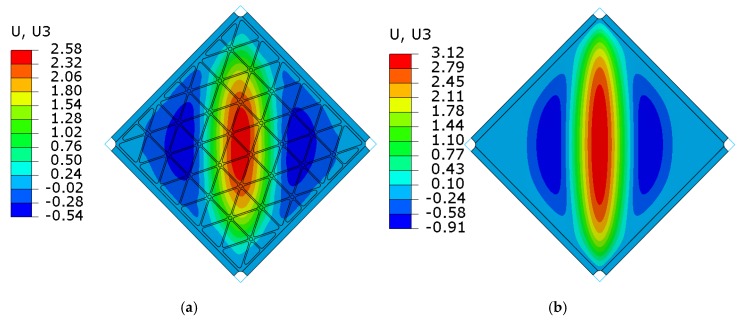
Deflection fields [mm] obtained from FEM analyses at the load level causing the start of plastic deformation (q = 37 N/mm): (**a**) stiffened plate, (**b**) smooth plate.

**Figure 23:**
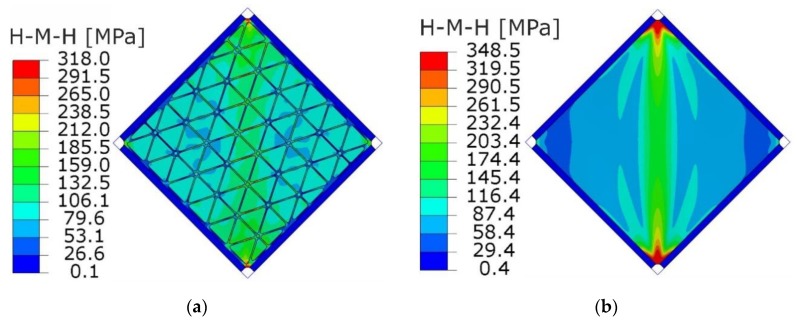
HMH stress distribution (MPa) obtained from FEM analyses at the load level causing the start of plastic deformation (q = 37 N/mm): (**a**) stiffened plate, (**b**) smooth plate.

**Figure 24:**
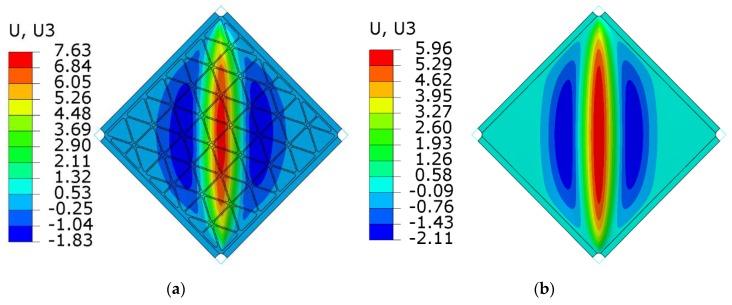
Deflection fields (mm) obtained from FEM analyses at the level of a maximum load equal to 103 N/mm: (**a**) stiffened plate, (**b**) smooth plate.

**Figure 25:**
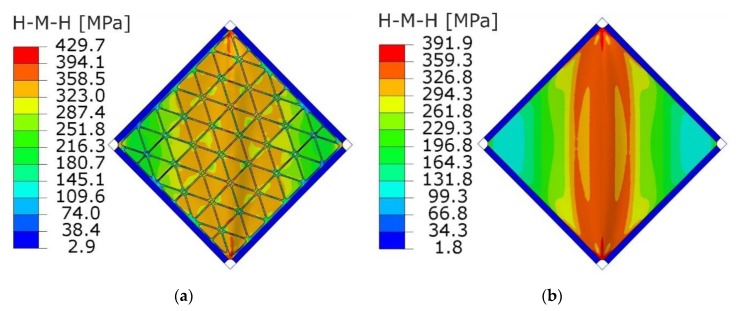
HMH stress distribution (MPa) obtained from FEM analyses at the level of load equal to 103 N/mm: (**a**) stiffened plate, (**b**) smooth plate.

Figure 20 and Figure 21 present the comparison of deflection lines of cross-sections passing through a horizontal diagonal for both plates for load levels equal to 37 N/mm and 103 N/mm. At the first value of loading, the stiffened plate exhibited the first plastic strain, and the second one was at the maximum load level. Deflection fields and Huber-Mises-Hencky (HMH) stress efforts for both mentioned load conditions and considered plates are presented in Figure 22, Figure 23, Figure 24 and Figure 25. It can be noted that the introduction of grid stiffening reduced deflection by 20%, with a corresponding decrease in stress by 9.6%, for the load level corresponding to initial permanent deformations of the structures.

As predicted by linear buckling analysis, both plates lost stability in the form of one dominant half-wave in the centre and two symmetric, smaller waves near the edge (Figure 17). Such deformations increased in magnitude with increasing load levels, but the overall shape did not change till the end of the analysis.

Similar to the graphs in Figure 19, deflections for the triangular, grid-stiffened plate were lower than for the smooth one only in the range of elastic deformations. The reason for such phenomena may be the high concentration of stress near the corners of the stiffened plate (Figure 23 and Figure 25). An increase in the stress gradient in these areas was probably connected with the method of FEM discretization of the structure as well as incorrect geometry of the structure, which can be treated as a design error.

## 7. Conclusions

The paper presents the results of experimental and numerical investigations of thin-walled plates stiffened by an isosceles grid pattern of low-profile ribs. The behaviour of the structure under pure shear loading conditions was considered in the range of nonlinear deformations, including post-buckling and plasticity effects. The main conclusions drawn are as follows:

(1) Introduction of triangularly arranged ribs increased the critical load by 300% in comparison to the smooth plate with equivalent mass. This phenomenon can be used in situations where it is intentional to maintain the shape of the structure (e.g., impact on aerodynamics), or directly when the local loss of stability is not permissible and critical load is equal to limit load.
(2) Comparisons of post-critical behaviours of the investigated structures proved that in the range of elastic deformation, the stiffened plate may have significantly better properties in the case of deflection and stress level, without an increase in the mass of the structure.
(3) Use of integral triangular stiffening in the design of thin-walled elements made it also possible to reduce the mass of the load-bearing structures maintaining the critical and limit load. This issue will be the subject of further work.
(4) Despite the use of certain simplifications covering both the geometry of the structure and the material model in the FEM analyses, it should be noted that results with a satisfactory degree of convergence were obtained.
(5) Reduced properties of the stiffened plate in the range of the plastic deformations are related to the stress concentrations. Such effects are probably caused by the incorrect geometry of the rib pattern around the corners of the plate, resulting in changes to the rigidity of the structure in the areas under consideration. More research should be carried out to obtain the methodology of the correct selection of geometry near the attachment areas. This fact should be taken into consideration when designing real construction solutions.
(6) Considering the occurrence of significant stress concentrations, it is necessary to carry out fatigue tests that will prove the impact of introducing the isosceles grid pattern of low-profile, integral ribs on the service life of the structure.

To summarize, the presented results make it possible to formulate the statement that integral, triangular sub-stiffening indicate a positive effect on the deformations and the stress state of thin-walled structures.

## Figures and Tables

**Table 1:** The basic nominal mechanical properties of materials used in the FEM model.

Parameter Name	Young’s Modulus E, GPa	Poisson Ratio	Ultimate Tensile Stress R_m_, MPa	Yield Stress R_p0.2_, MPa
Al2024	70	0.33	488	345
Steel	210	0.3	-	-

## Supplementary Materials

The following are available online at https://www.mdpi.com/1996-1944/12/22/3699/s1, Video S1: Deformations of the stiffened plate captured by the DIC system.
